# Effectiveness of an AI-Assisted Digital Workflow for Complete-Arch Implant Impressions: An In Vitro Comparative Study

**DOI:** 10.3390/dj13100462

**Published:** 2025-10-09

**Authors:** Marco Tallarico, Mohammad Qaddomi, Elena De Rosa, Carlotta Cacciò, Silvio Mario Meloni, Ieva Gendviliene, Wael Att, Rim Bourgi, Aurea Maria Lumbau, Gabriele Cervino

**Affiliations:** 1 Department of Medicine, Surgery and Pharmacy, University of Sassari, 07100 Sassari, Italy; smeloni@uniss.it (S.M.M.); alumbau@uniss.it (A.M.L.); 2 Department of Esthetic and Prosthetic Dentistry, School of Dentistry, Saint-Joseph University of Beirut, Beirut 1107 2180, Lebanon; mohammadqaddomi15@gmail.com; 3 School of Dentistry, University of Sassari, 07100 Sassari, Italy; elenaderosa0@gmail.com (E.D.R.); carlotta.caccio@gmail.com (C.C.); 4 Faculty of Medicine, Institute of Odontology, Vilnius University, Zalgirio g. 117, LT-08217 Vilnius, Lithuania; ieva.gendviliene@gmail.com; 5 Department of Prosthetic Dentistry, University Medical Center of Freiburg, 79106 Freiburg, Germany; waelatt@gmail.com; 6 Private Practice, The Face Dental Group, Boston, MA 02215, USA; 7 Department of Restorative Dentistry, School of Dentistry, Saint-Joseph University, Beirut 1107 2180, Lebanon; rim.bourgi@hotmail.com; 8 Department of Biomaterials and Bioengineering, INSERM UMR_S 1121, University of Strasbourg, 67000 Strasbourg, France; 9 Department of Restorative Sciences, Faculty of Dentistry, Beirut Arab University, Beirut 115020, Lebanon; 10 Department of Biomedical and Dental Sciences and Morphofunctional Imaging, University of Messina, 98168 Messina, Italy

**Keywords:** digital workflow, artificial intelligence, SmartX, intraoral scanner, implant dentistry, Complete-Arch impressions

## Abstract

**Background**: The accuracy and consistency of complete-arch digital impressions are fundamental for long-term success of implant-supported rehabilitations. Recently, artificial intelligence (AI)-assisted tools, such as SmartX (Medit Link v3.4.2, MEDIT Corp., Seoul, South of Korea), have been introduced to enhance scan body recognition and data alignment during intraoral scanning. **Objective**: This in vitro study aimed to evaluate the impact of SmartX on impression accuracy, consistency, operator confidence, and technique sensitivity in complete-arch implant workflows. **Methods**: Seventy-two digital impressions were recorded on edentulous mandibular models with four dummy implants, using six experimental subgroups based on scan body design (double- or single-wing), scanning technique (occlusal or combined straight/zigzag), and presence/absence of SmartX tool. Each group was scanned by both an expert and a novice operator (n = 6 scans per subgroup). Root mean square (RMS) deviation and scanning time were assessed. Data were tested for normality (Shapiro–Wilk). Parametric tests (*t*-test, repeated measures ANOVA with Greenhouse–Geisser correction) or non-parametric equivalents (Mann–Whitney U, Friedman) were applied as appropriate. Post hoc comparisons used Tukey HSD or Dunn–Bonferroni tests (α = 0.05). **Results**: SmartX significantly improved consistency and operator confidence, especially among novices, although it did not yield statistically significant differences in scan accuracy (*p* > 0.05). The tool mitigated early scanning errors and reduced dependence on operator technique. SmartX also enabled successful library alignment with minimal data; however, scanning time was generally longer with its use, particularly for beginners. **Conclusions**: While SmartX did not directly enhance trueness, it substantially improved scan reliability and user experience in complete-arch workflows. Its ability to minimize technique sensitivity and improve reproducibility makes it a valuable aid in both training and clinical settings. Further clinical validation is warranted to support its integration into routine practice.

## 1. Introduction

Modern dentistry is undergoing a paradigm shift driven by the progressive adoption of digital technologies that span diagnostics, surgical planning, prosthetic design, and clinical execution. Among the most impactful innovations is computer-guided implantology, which has become a cornerstone in contemporary treatment protocols—particularly in complex scenarios such as aesthetic zone management and full-arch rehabilitations [1]. This approach enhances the predictability and safety of surgical procedures, minimizes complications, improves patient experience, and increases clinical efficiency [2].

The synergistic integration of cone beam computed tomography (CBCT), intraoral scanning (IOS), and advanced digital planning software has empowered clinicians to simulate treatments virtually and translate them with high fidelity into operative procedures. Guided surgery, supported by 3D-printed templates, enables precise implant placement consistent with prosthetically driven planning [3]. However, despite these technological advancements, full-arch implant rehabilitations remain among the most challenging procedures in implant dentistry due to the need to restore function, esthetics, and longevity in patients who often lack reliable anatomical reference points.

Digital workflows offer a promising solution by providing individualized planning and real-time intraoperative control, thus improving overall treatment predictability. In particular, IOS devices allow for rapid, non-invasive acquisition of intraoral geometry with accuracy levels that now rival or even surpass those of traditional impression techniques [4,5]. When integrated with CBCT data, these scans create a comprehensive virtual representation of the patient’s anatomy—a so-called “digital twin”—which can be utilized across all treatment phases, from diagnosis to prosthetic delivery and long-term maintenance.

Artificial Intelligence (AI) represents a new frontier in this transformation. AI-based systems are capable of processing large datasets to support clinical decision-making, automate repetitive tasks, and enhance the consistency of outcomes. In implant dentistry, AI contributes to more accurate diagnosis, optimized implant positioning, and real-time analysis of biomechanical variables [6,7]. These benefits translate into less invasive procedures, improved prosthetic integration, and potential for immediate loading protocols.

Nonetheless, the clinical success of implant treatments remains multifactorial. Beyond digital integration, outcomes depend on implant macro- and micro-design, surgical expertise, and soft tissue management. For example, implants with enhanced surface hydrophilicity have demonstrated superior osseointegration and better support for immediate loading [8]. Furthermore, clinicians must possess a solid understanding of software workflows, scan strategies, and the biomechanical management of edentulous patients to fully exploit the potential of digital tools.

In this context, guided implant surgery supported by 3D-printed templates facilitates a highly controlled surgical environment, minimizing deviations and tissue trauma [9]. Each step of the workflow—from the initial impression to guide fabrication, implant insertion, and final prosthesis delivery—requires high accuracy and consistency. Effective collaboration with dental laboratories, made possible through digital communication channels, enables the design and production of prostheses through computer-aided design and manufacturing (CAD/CAM), reducing human error and enhancing treatment quality.

The integration of CBCT, IOS, AI, and 3D printing is thus redefining implantology into a more predictive, preventive, and personalized model of care. Complete-arch rehabilitations exemplify this evolution, showcasing how digital dentistry can provide highly accurate and patient-centered solutions [10]. However, despite the promising potential, full-arch IOS in edentulous cases is still limited by the lack of stable anatomical landmarks, frequent scan body recognition errors, and variability introduced by operator technique—all of which may compromise accuracy and reproducibility [11].

Recent studies have evaluated segmental scan strategies and data merging techniques in an effort to overcome these challenges, with mixed results [11]. In light of these limitations, new tools that incorporate AI-assisted guidance and enhanced scan body designs may offer a path forward. Specifically, SmartX (Medit Link v3.4.2, MEDIT Corp., Seoul, Republic of Korea) integrates AI algorithms to improve scan body recognition and library alignment in real time, potentially compensating for incomplete data and minimizing operator-related inconsistencies.

Therefore, the present in vitro study was designed to assess whether SmartX, in combination with a novel scan body design featuring lateral extensions, can improve the precision and reproducibility of full-arch digital impressions. The specific aim was to validate this AI-supported impression workflow for all-on-X implant rehabilitations. Accordingly, two null hypotheses were tested: (H_01_) there is no significant difference in impression accuracy (measured by root mean square deviation, RMS) between an expert and a novice operator; and (H_02_) there is no significant difference in accuracy between different scanning techniques.

## 2. Materials and Methods

### 2.1. Study Design

This in vitro study was conducted as a comparative analysis to evaluate the accuracy and precision of a novel digital workflow for complete-arch restorations, named SmartX (MEDIT Corp., Seoul, Republic of Korea). All the digital impressions were taken by two operators: a final-year dental student (FDR) and an experienced clinician with over two decades of practice in digital dentistry (MT). Prior to data collection, the dental student received structured training in digital impression techniques. This included a theoretical overview, a live demonstration, and hands-on practice with the scanner under the supervision of the MT, who also performed the comparative scans to ensure consistency. In addition, the same student participated in a previously reported paper on the accuracy and precision of digital impressions using reverse scan body (RSB) prototypes and IOSs for rehabilitating fully edentulous patients [12].

### 2.2. Sample Preparation and Group Allocation

The present study utilized mandible models replicating full edentulism with simulated gingiva, specifically manufactured for implantology training purposes. These models were designed to emulate D2 bone density, incorporating dense cortical and porous trabecular structures (Dentalstore & Edizioni Lucisano SRL, Milan, Italy). A CBCT scan (Cranex 3Dx, Soredex, Tuusula, Finland) was acquired using parameters of 90 kV and 5.0 mA, with a 6 × 8 field of view and 0.2 mm resolution. The Digital Imaging and Communications in Medicine (DICOM) data from the CBCT was subsequently superimposed with Standard Tessellation Language (STL) files generated via an optical scan (i900, Medit Corp., Yeongdeungpo-gu, Seoul, Republic of Korea) of the same model. A virtual diagnostic wax-up was created to guide prosthetic planning and implant positioning using dedicated planning software (exocad’s DentalCAD 3.2 Elefsina, exocad GmbH, Darmstadt, Germany). Virtual placement of four Osstem TSIII implants (4 mm diameter × 10 mm length; Osstem Implant Co., Ltd., Seoul, Republic of Korea) was performed using the same software, following the established protocol by Malò et al. [13]. An additional three buccal anchor pins were planned to secure the surgical guide during implant site preparation and insertion. A fully 3D-printed surgical template, designed without metal sleeves due to compatibility with the OneGuide Kit (Osstem Implants, Seoul, Republic of Korea), was fabricated at a specialized center (New Ancorvis SRL, Bologna, Italy) using a Diret Metal Printing (DMP) Dental 100 printer and certified resin material (VisiJet M2R-CL, 3D Systems Inc., Rock Hill, SC, USA). Four dummy implants were then fully guided using a surgical template without metallic sleeves and a dedicated surgical kit (ONEGuide, Osstem Global Co., Ltd., Seoul, Republic of Korea), adhering strictly to the manufacturer’s specifications. Following implant placement, multi-unit abutments and temporary cylinders (Osstem Implant Co., Ltd.) were secured using the recommended torque settings. Finally, a temporary restoration was rebased onto the temporary cylinders (Osstem Implant Co., Ltd.) using a resin cement (Panavia SA, Kuraray Europe GmbH, Hattersheim, Germany), with the aid of a screw-retained module attached to the base of the template, to guide the temporary restoration at the correct vertical dimension of occlusion and centric relation. After implant placement, digital impressions were taken. Both operators recorded the digital impressions across multiple groups, including the control group and various test subgroups.

**Figure 1:**
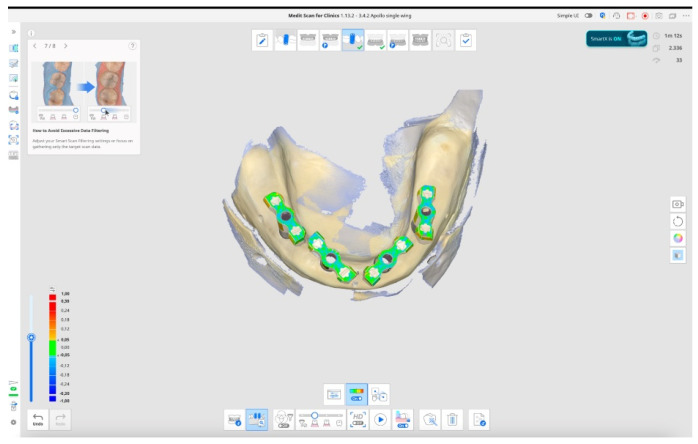
Test group impression using Medit i900 and Medit Link (version 3.4.2., MEDIT Corp., Seoul, Republic of Korea) software, with activated SmartX tool, and one scan impression technique using Scan bodies featured with double-wing lateral extensions (SmartFlags, Apollo, Poland). Performed movements: straight and zigzag motion in anterior, straight motion in posterior.

**Figure 2:**
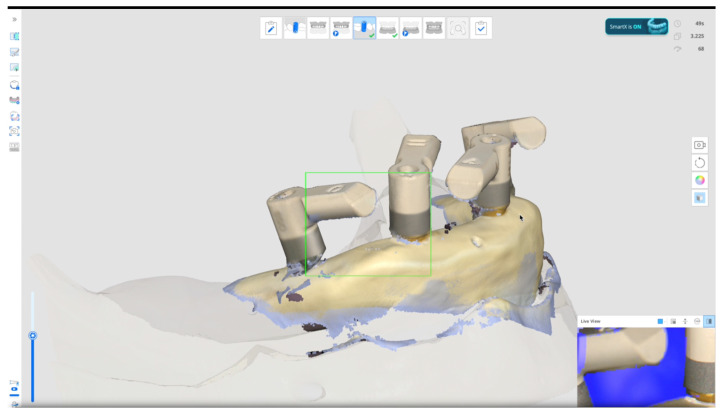
Test group impression using Medit i900 and Medit Link (version 3.4.2., MEDIT Corp., Seoul, Republic of Korea) software, with **NO** SmartX tool, and one scan impression technique using Scan bodies featured with single-wing lateral extensions (SmartFlags, Apollo, Poland). Performed movements: straight and zigzag motion in anterior, straight motion in posterior.

- In the **control group,** a first scan of the temporary restoration was taken. After that, the temporary restoration was unscrewed, four scan bodies (Osstem Implant Co., Ltd.) were torqued at 15 Ncm using the E-Driver (Osstem Implant Co., Ltd.), and a second digital impression was recorded. Both scans were taken using a desktop scanner (Nobil Metal SPA, Villafranca D’Asti, Italy) to serve as a reference to evaluate accuracy and precision of the other test groups.
- In the **test group**, six subgroups were created, with six repeated scans taken for each subgroup, resulting in a total of 36 scans performed by the expert operator and another 36 by the student. All the digital scans were taken with Medit i900 (Medit Corp., Seoul, Republic of Korea) scan onto the four multi-unit abutments. Subgroups are divided by scan body design/scan technique/operator/as follows:
  - Scan bodies featured with double-wing lateral extensions (SmartFlags, Apollo, Poland)/Occlusal scan/straight motion/SmartX tool/Expert (n = 6), Student (n = 6). An example is reported in Figure 1.
  - Scan bodies featured with double-wing lateral extensions (SmartFlags, Apollo, Poland)/Occlusal scan/straight motion/No SmartX tool/Expert (n = 6), Student (n = 6).
  - Scan bodies featured with double-wing lateral extensions (SmartFlags, Apollo)/One scan/straight and zigzag motion in anterior, straight motion in posterior/SmartX tool/Expert (n = 6), Student (n = 6).
  - Scan bodies featured with double-wing lateral extensions (SmartFlags, Apollo)/One scan/straight and zigzag motion in anterior, straight motion in posterior/NO SmartX tool/Expert (n = 6), Student (n = 6).
  - Scan bodies featured with single-wing lateral extension (SmartFlags, Apollo)/One scan/straight and zigzag motion in anterior, straight motion in posterior/SmartX tool/Expert (n = 6), Student (n = 6).
  - Scan bodies featured with single-wing lateral extension (SmartFlags, Apollo)/One scan/straight and zigzag motion in anterior, straight motion in posterior/NO SmartX tool/Expert (n = 6), Student (n = 6). An example in Figure 2.

Subgroups at the test group are summarized in Table 1.

### 2.3. Outcome Measures

The outcome measures were the accuracy and precision of the impressions, along with the operative time required to record impressions.

**Figure 3:**
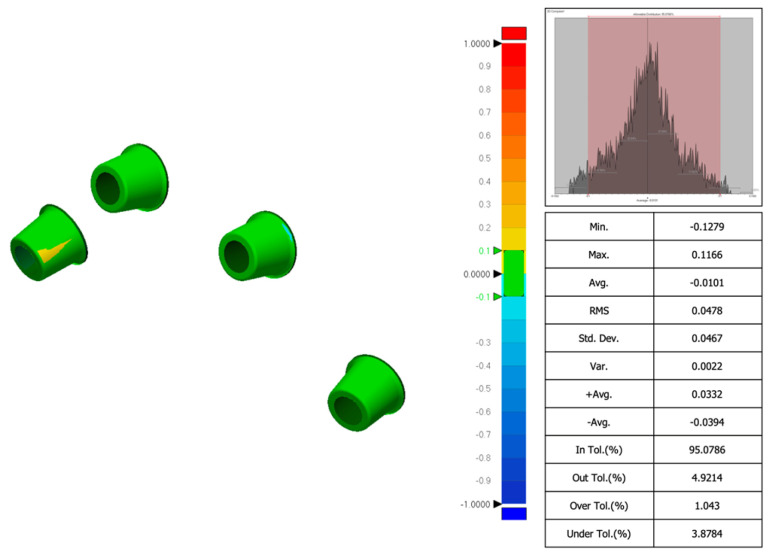
Root mean square (RMS) deviation analysis performed using Geomagic Control X software (version 2022.1.0; 3D Systems, Rock Hill, SC, USA), comparing intraoral scan data with reference models for accuracy evaluation.

Accuracy was defined as the degree of conformity between the captured data and the actual anatomical dimensions, while precision referred to the consistency of repeated measurements. Accuracy depended on appropriate device calibration and software optimization. Comparisons between the tested groups were performed with superimposition of the files. The STL files obtained from both intraoral scanner and desktop scanners were imported into a dental CAD software (Exocad 3.1 Rijeka prototype, Exocad GmbH, Darmstadt, Germany). Scan bodies were digitally aligned with their corresponding library components to analyze factors affecting accuracy. The basal surfaces of the abutments, considered non-confidential parts of the implant system, were then exported in STL format for evaluation (Figure 2). To assess scan accuracy, dimensional deviations were quantified using the Root Mean Square (RMS) value derived from 3D superimpositions. All scan files were subsequently analyzed in Geomagic Control X (version 2022.1.0, 3D Systems, Rock Hill, SC, USA), where they were compared to the reference dataset (control group) to determine any dimensional differences (an example in Figure 3). Particularly, 3D deviation across surface meshes (STL files of planned versus actual implant positions) was compared, and a color-coded deviation map was generated. Operative time was measured as the total time in seconds required to obtain an impression. Timing began at the start of scanning and ended with either scan completion. All measurements were carried out by an experienced examiner (MQ). Geomagic software does not require direct calibration; scan-to-scan comparisons were validated using a control scan as reference (control group).

### 2.4. Statistical Analysis

All datasets were first tested for normality using the Shapiro–Wilk test. When data followed a normal distribution, parametric tests were applied; otherwise, non-parametric equivalents were used. For between-group comparisons (expert vs. student operators), we used the independent samples *t*-test (parametric) or the Mann–Whitney U test (non-parametric). For within-group comparisons (different scanning techniques and conditions performed by the same operator), we employed a repeated measures ANOVA. In case the assumption of sphericity was violated (tested with Mauchly’s test), the Greenhouse–Geisser correction was applied. When normality assumptions were not met, the Friedman test was used as a non-parametric alternative. Post hoc pairwise comparisons were carried out using the Tukey Honestly Significant Difference (HSD) test for ANOVA, or the Dunn–Bonferroni test for Friedman, with adjustment for multiple testing. All tests were two-tailed, with the significance level set at α = 0.05. Statistical analysis was performed using SPSS v.29.0 (IBM Corp., Armonk, NY, USA).

## 3. Results

In the test group, 72 scans were taken in six subgroups (6 each in both student and expert). However, occlusal scan with straight motion without AI tool (no SmartX) failed to generate complete scanned data around the scan body in both the expert and student. Consequently, matching with the reference libraries was not performed. For this reason, only 60 impressions were measured and compared. On the contrary, using SmartX tool and double-wing scan bodies (SmartFlags, Apollo, Poland) with one occlusal/straight motion, the mean RMS value was 0.0670 ± 0.0238 when scanned by the expert in digital dentistry, while it was 0.0634 ± 0.0082 when scanned by the student. The difference was not statistically significant (*p* = 0.732).

Using SmartX tool and double-wing scan bodies (SmartFlags, Apollo, Poland) with one occlusal/straight motion, the mean RMS value was 0.0670 ± 0.0238 when scanned by the expert in digital dentistry, while it was 0.0634 ± 0.0082 when scanned by student. The difference was not statistically significant (*p* = 0.736). Adding Zigzag movement to the straight movement, the accuracy increases with a mean RMS of 0.0540 ± 0.0104 (expert) and 0.0586 ± 0.0152 (student). The difference was not significant (0.0047 ± 0.0156; *p* = 0.548). Similar results were found using SmartX/Single-wing and One/Straight/Zigzag technique. Comparing expert and student, the first null hypothesis (H0) could not be rejected. In other words, using the SmartX tool, the difference in RMS between expert and student is not big enough to be statistically significant. Data are reported in Table 2.

Since the *p*-value was *p* > 0.05, the second null hypothesis (H0) could not be rejected. In other words, the scanning technique does not seem to influence the final accuracy when the SmartX tool is used. In addition, within-group analyses failed to find statistically significant differences. In the expert group, the f-ratio value was 1.09793 and the *p*-value was 0.379298, while in the student group, the f-ratio value was 1.63283 and the *p*-value was 0.197231. In both groups, the results were not significant at *p* < 0.05. However, occlusal scan with straight motion without AI tool (no SmartX) failed to generate good impression to be measured, but not when the SmartX tool was used. In other words, SmartX improved accuracy and confidence in this case. In addition, comparing the four single-wing scan bodies and occlusal scan (straight motion), with the same scan bodies and one scan (straight and zigzag motion in anterior, straight motion in posterior), the difference was statistically significant in the student (*p* = 0.049) but not in the expert (*p* = 0.383). In other words, SmartX and single-wing can be easier to scan for beginners. All the data are reported in Table 2.

Comparing the time, except that for the occlusal/straight digital impression, in all the other digital impressions, the time needed to record the impression was lower for the expert compared with the student. A trend of lower time was found in both student and expert for the double-wing/one/straight and zigzag impression (combined). On the contrary, a trend toward longer scanning time was found in case of single-wing scan bodies. All the data are reported in Table 3.

## 4. Discussion

This in vitro study evaluated the effectiveness of a novel digital impression workflow integrating artificial intelligence (AI) through the SmartX tool (Medit Link v3.4.2, MEDIT Corp., Republic of Korea) in combination with innovative scan body designs featuring lateral extensions. The goal was to assess whether this approach could enhance the accuracy, consistency, and operator confidence in full-arch implant impressions—a well-recognized clinical challenge due to the absence of stable anatomical landmarks in edentulous arches.

The findings demonstrated that SmartX significantly improved the reproducibility of impressions and operator confidence, particularly among novice users. While the tool did not lead to a statistically significant increase in impression accuracy, it consistently minimized technique sensitivity and facilitated successful library alignment even in cases of incomplete scan data. This suggests that SmartX contributes more to workflow stability and reliability rather than to intrinsic trueness of the scan.

Notably, the use of scan bodies with lateral extensions—especially in conjunction with SmartX—yielded high precision across both operators and scanning techniques. Conversely, when SmartX was not employed, occlusal scans with a straight motion failed to produce clinically usable impressions. This reinforces the importance of intelligent guidance tools in managing the complex geometry of full-arch cases. The improved performance of combined straight and zigzag scanning patterns further supports the adoption of dynamic scan strategies in digital protocols.

The clinical relevance of these findings lies in the increasing demand for full-arch restorations that combine precision, efficiency, and patient-centered care [14]. Achieving a passive fit of the definitive prosthesis remains a fundamental requirement for long-term success, as it minimizes biomechanical stress on implants and peri-implant tissues, reducing the risk of complications such as screw loosening, framework fractures, and peri-implantitis [11,15,16,17]. In this study, the combination of multi-unit abutments, IOS, and CAD/CAM-fabricated metal frameworks reflected a clinically replicable scenario where digital precision directly impacts prosthetic fit—validated by established protocols such as the Sheffield (one-screw) test.

Accurate three-dimensional positioning of implants is another cornerstone of successful outcomes. In the edentulous maxilla, anatomical variability and mucosal mobility pose additional challenges. Fully guided surgery using pin-retained, 3D-printed templates—as applied in this study—not only enhanced placement accuracy but also facilitated adjunctive procedures such as minimally invasive sinus lifts [18,19,20]. The ability to convert digital plans into surgical precision further underscores the value of an integrated digital ecosystem that includes CBCT, IOS, and AI-enhanced impression capture [21,22].

To the best of our knowledge, this is the first study to systematically investigate the performance of an AI-based impression tool like SmartX in a controlled, full-arch implant setting. The lack of comparable studies in the literature precludes direct benchmarking, but the results point toward a clear benefit in reducing inter-operator variability and enhancing user experience. This is particularly relevant in clinical education and training environments, where novice operators often struggle with mastering intraoral scanning techniques.

In addition, the SmartX protocol demonstrated encouraging trends regarding impression time. Occlusal scans with straight motion were the fastest (approximately 20 s for four implants), but they were only effective when SmartX was used. Combined scan paths (straight and zigzag) in conjunction with double-wing scan bodies showed the best balance between speed and precision. Conversely, single-wing scan bodies required longer acquisition times, likely due to reduced surface features for progressive image stitching.

One noteworthy technical observation involved the failure of scan stitching in occlusal straight scans without SmartX. This likely resulted from the scanner’s inability to register consistent reference points in the absence of AI-driven alignment, especially when encountering data voids. In such cases, the scanner software may attempt to interpolate surfaces based on adjacent morphology, which—if insufficient—leads to artifacts such as bulging or hollow distortions. The SmartX tool appeared to mitigate this issue by anchoring scan body recognition and guiding real-time alignment, even with partial datasets.

Although objective metrics such as RMS values and scan time were the focus of this study, the perceived ease of use and operator confidence associated with SmartX should not be overlooked. These subjective aspects are increasingly recognized as critical factors in the adoption of digital tools. Future research should incorporate validated instruments such as the System Usability Scale (SUS), NASA Task Load Index (NASA-TLX), and the User Experience Questionnaire (UEQ) to assess usability, workload, learning curve, and overall satisfaction. Such data would be especially valuable in multicenter or educational contexts, helping to benchmark operator experiences and streamline digital workflow implementation across various clinical settings. In summary, while SmartX may not replace foundational scanning principles or compensate for all sources of error, it clearly enhances digital workflow robustness, particularly for less experienced users. Its integration into full-arch protocols represents a meaningful advancement toward standardized, efficient, and operator-friendly implant dentistry.

This study highlights the promising role of AI-enhanced digital workflows in improving the consistency and user confidence associated with complete-arch implant impressions. The integration of SmartX with guided surgery and CAD/CAM fabrication allowed for a streamlined, accurate, and reproducible workflow, particularly beneficial in scenarios where operator skill level or anatomical complexity may otherwise compromise outcomes. While the SmartX tool did not significantly improve the trueness of digital impressions in terms of root mean square (RMS) deviation, it clearly enhanced scan completeness and reduced technique sensitivity—especially among less experienced operators. These findings underscore its potential value as both a clinical aid and an educational support tool within digital implantology.

Nevertheless, several limitations must be acknowledged. First, the study was conducted in an in vitro setting using standardized edentulous mandibular models with an all-on-four configuration. This experimental design, while controlled, may not fully replicate the complexities of real-life clinical conditions, such as intraoral humidity, patient movement, limited accessibility, or varying soft tissue morphology. For these reasons, the results of the present research can not be directly transferred to clinical situations. Second, the small sample size (72 planned scans) and the involvement of only two operators limit the statistical power and generalizability of the findings. Broader validation across multiple operators, centers, and patient populations is required to confirm the reproducibility and clinical relevance of these results. Moreover, this study exclusively evaluated a single type of intraoral scanner (Medit i900) and a limited range of scan body geometries. Therefore, the results of this study are not easily generalizable and mainly highlight the need for future multi-scanner studies. Additional research incorporating a wider variety of IOS systems and commercially available scan body designs could offer a more comprehensive understanding of SmartX’s performance and its applicability across different digital platforms. Another area of limitation relates to the subjective evaluation of operator confidence. While observationally noted, these impressions were not quantified using standardized usability instruments. Future studies should include validated assessment tools—such as the System Usability Scale (SUS), NASA Task Load Index (NASA-TLX), and User Experience Questionnaire (UEQ)—to objectively measure learning curve, user satisfaction, and perceived workload. Importantly, the failure of occlusal scans with straight motion in the absence of SmartX underscores the need to further investigate the limitations of current image stitching algorithms, particularly their reliance on consistent morphological reference points. The development of more adaptive, machine-learning-based scanning protocols that respond in real time to user behavior and intraoral conditions may represent the next step in improving the robustness of digital impressions.

## 5. Conclusions

The SmartX tool does not replace foundational scanning principles; however, it significantly improves the reliability and reproducibility of digital impressions in complete-arch workflows—especially in the hands of novice users. Further clinical investigations in diverse real-world settings are warranted to validate these results and support broader integration into routine practice.

## Figures and Tables

**Table 1:** Test group: subgroups.

Subgroup	SB * Design	Technique	SmartX	Expert	Student	Total
**Double-wing/Occlusal/SmartX**	Double-wing	1	Yes	6	6	12
**Double-wing/Occlusal/No SmartX**	Double-wing	1	No	6	6	12
**Double-wing/Combined Scan/SmartX**	Double-wing	2	Yes	6	6	12
**Double-wing/Combined Scan/No SmartX**	Double-wing	2	No	6	6	12
**Single-wing/Combined Scan/SmartX**	Single-wing	2	Yes	6	6	12
**Single-wing/Combined Scan/No SmartX**	Single-wing	2	No	6	6	12
**Grand total**				36	36	72

* SB: Scan body; 1 Occlusal scan/straight. 2 One Scan/Straight and zigzag motion in anterior/straight in posterior.

**Table 2:** Root mean square (RMS) values (mm) within subgroups.

	SmartX/Double-Wing Occlusal/Straight	NoSmartX/Double-Wing One/Straight/Zigzag	SmartX/Double-Wing One/Straight/Zigzag	NoSmartX/Single-Wing One/Straight/Zigzag	SmartX/Single-Wing One/Straight/Zigzag
**Expert**	0.0670 ± 0.0238	0.0547 ± 0.0044	0.0540 ± 0.0104	0.0578 ± 0.0030	0.0575 ± 0.0060
**Student**	0.0634 ± 0.0082	0.0515 ± 0.0054	0.0586 ± 0.0152	0.0570 ± 0.0046	0.0545 ± 0.0043
**Difference**	0.0036 ± 0.0249	0.0032 ± 0.0079	0.0047 ± 0.0156	0.0008 ± 0.0055	0.0030 ± 0.0093
***p* Value**	0.736	0.282	0.548	0.734	0.346

Statistical tests Table 2: independent samples *t*-test (parametric) or Mann–Whitney U test (non-parametric) depending on data distribution.

**Table 3:** Digital impression time in seconds within subgroups.

	SmartX/Double-Wing Occlusal/Straight	NoSmartX/Double-Wing One/Straight/Zigzag	SmartX/Double-Wing One/Straight/Zigzag	NoSmartX/Single-Wing One/Straight/Zigzag	SmartX/Single-Wing One/Straight/Zigzag
**Expert**	21.3 ± 3.1	70.5 ± 2.2	68.8 ± 1.9	66.7 ± 2.4	67.8 ± 2.3
**Student**	22.5 ± 1.0	75.8 ± 2.2	74.7 ± 2.6	73.3 ± 2.3	77.0 ± 2.1
**Difference**	1.2 ± 3.9	5.3 ± 1.0	4.8 ± 3.4	6.7 ± 3.8	9.2 ± 7.8
***p* Value**	0.412	0.002	0.005	0.012	0.001

Statistical tests Table 3: independent samples *t*-test (parametric) or Mann–Whitney U test (non-parametric) depending on data distribution.

## Data Availability

The original contributions presented in the study are included in the article, further inquiries can be directed to the corresponding authors.
